# Green Synthesized ZnO Nanoparticles as Biodiesel Blends and Their Effect on the Performance and Emission of Greenhouse Gases

**DOI:** 10.3390/molecules27092845

**Published:** 2022-04-29

**Authors:** Kiran Kavalli, Gurumoorthy S. Hebbar, Jayachamarajapura Pranesh Shubha, Syed Farooq Adil, Mujeeb Khan, Mohammad Rafe Hatshan, Adibah Mukhlid Almutairi, Baji Shaik

**Affiliations:** 1 Department of Mechanical & Automobile Engineering, Christ University, Bangalore 560029, India; kiran.k@christuniversity.in (K.K.); gurumoorthy.hebbar@christuniversity.in (G.S.H.); 2 Department of Chemistry, Don Bosco Institute of Technology, Mysore Road, Bangalore 560074, India; 3 Department of Chemistry, College of Science, King Saud University, P.O. Box 2455, Riyadh 11451, Saudi Arabia; kmujeeb@ksu.edu.sa (M.K.); mhatshan@ksu.edu.sa (M.R.H.); adeba@ksu.edu.sa (A.M.A.); 4 Department of Advanced Materials Engineering for Information & Electronics, Kyung Hee University, 1732 Deogyeong-daero, Giheung-gu, Yongin-si 446-701, Gyeonggi-do, Korea; shaikbaji2@khu.ac.kr

**Keywords:** ZnO nanoparticles, green synthesis, cow dung, biodiesel, greenhouse gases

## Abstract

Pollution and global warming are a few of the many reasons for environmental problems, due to industrial wastes and greenhouse gases, hence there are efforts to bring down such emissions to reduce pollution and combat global warming. In the present study, zinc oxide nanoparticles are green synthesized using cow dung as fuel, through combustion. Synthesized material was characterized by FTIR, XRD, UV, and FESEM. The as-prepared ZnO-GS NPs were employed as a transesterification catalyst for the preparation of biodiesel from discarded cooking oil. The biodiesel obtained is termed D-COME (discarded cooking oil methyl ester), which is blended with 20% commercial diesel (B20). Additionally, this blend, i.e., B20, is further blended with varying amounts of as-prepared ZnO-GS NPs, in order to ascertain its effects on the quality of emissions of various greenhouse gases such as hydrocarbons, CO_x_, NO_x_. Moreover, the brake thermal efficiency (BTHE) and brake specific fuel consumption (BSFC) were studied for their blends. The blend (B20) with 30 mg of ZnO-GS, i.e., B20-30, displays the best performance and reduced emissions. Comparative studies revealed that the ZnO-GS NPs are as efficient as the ZnO-C NPs, indicating that the green synthetic approach employed does not affect the efficiency of the ZnO NPs.

## 1. Introduction

The world is presently confronted with the twin crises of fossil fuel depletion and environmental degradation [1]. Indiscriminate extraction and lavish consumption of fossil fuels have led to a reduction in underground-based carbon resources [2]. Aside from this, gasoline and diesel-driven automobiles are major sources of greenhouse gas (GHG) emissions. Therefore, the search for alternative fuels, which promise a harmonious correlation with sustainable development, energy conservation, efficiency, and environmental preservation, has become a pressing matter in the present situation [3]. In this context, fuels of bio-origin can provide a feasible solution to this worldwide petroleum crisis. Recently, biodiesel has gained tremendous popularity as an efficient and environmentally friendly alternative to fossil fuels. 

Biodiesel consists of lower alkyl ester(s) of the long-chain fatty acids and is mainly produced by the transesterification of vegetable oils or by the esterification of free fatty acids with lower alcohols. It is considered an eco-friendly fuel, due to its low emission profile, non-toxicity, and biodegradability, which also helps in controlling global warming. Usually, biodiesel is produced from lower alkyl esters of vegetable oils (via transesterification), such as rapeseed oil, soya bean oil, etc.; however, the heavy consumption and high cost of these raw materials have made biodiesel costly, which seriously affected its large-scale production. To control the price, researchers have focused on non-edible oils which are not suitable for human consumption and can be easily obtained from the plants which grow in barren lands. Recently, among various non-edible oils, waste cooking oil (WCO) has gained a decent amount attention due to its renewable nature and easy availability. Moreover, WCO is usually discarded in drains and the increasing number of restaurants creates huge disposal problems. Therefore, the application of WCO to produce biodiesel offers an effective approach for the handling of cooking waste while contributing to the global energy demand.

Transesterification is the reaction of glycerides of oils with an alcohol in the presence of an enzymatic acid/base homogenous or heterogeneous catalyst (which increases the rate of reaction and yield) to produce esters and glycerol. Among various alcohols, methanol and ethanol are widely used, while the former is preferred due to its low cost. Homogeneous catalysts including NaOH and KOH are commonly used during transesterification reactions; however, they are non-recoverable, corrosive, and produce high amounts of soap and by-products. On the other hand, heterogeneous catalysts are relatively more beneficial due to their high activity, reusability stability, easy regeneration, and high water tolerance. Aside from this, heterogeneous catalysts can perform both esterification and transesterification simultaneously [4]. So far, a variety of heterogeneous catalysts have been used for the production of biodiesel, including zeolites, metal oxides, hydrotalcite, etc. Preference for metal oxides over other materials has been increasing due to their low-cost, easy availability, high basicity, and enhanced reusability [5]. In addition, metal oxide nanomaterials offer enhanced surface area, which ultimately increases the catalytic activity of the material by increasing the basic nature of the catalyst [6].

Recently, among various metal oxides, the idea of the application of zinc oxide nanoparticles (ZnO NPs) as catalysts for the esterification of free fatty acids (FFAs) and the transesterification of triglycerides has gradually gained traction among researchers. ZnO is an amphoteric transition-metal oxide which has been used as a heterogeneous catalyst for various catalytic conversions, including the conversion of various types of edible feedstock, including rapeseed oil, palm oil, and soybean oil, to biodiesel [7,8,9]. However, only a few studies have reported on the application of ZnO NPs as heterogeneous catalysts for the conversion of non-edible feedstock including WCO to biodiesel [10]. ZnO NPs can be obtained from various synthetic approaches, such as coprecipitation, colloidal, sol-gel processing, water-oil microemulsions, hydrothermal, solvothermal, sonochemical, and polyol methods, etc. [11]. However, most of these techniques typically require a long time, high temperatures, tedious procedures, or the use of lengthy procedures, resulting in laborious operations [12]. Among available techniques for the preparation of ZnO NPs, the solution combustion method is facile, quick, economic, easy to scale up without the help of special ligands, and capable of producing a pure crystalline product [13].

Even though biodiesel has engrossed recent research, it also has certain limitations, such as the high emission of NO_x_, less energy output, and more fuel consumption [14]. To overcome these shortcomings, biodiesel properties are usually modified by the inclusion of high-energy nano additive contents [15]. So far, a variety of studies have reported on the study of the effect of nano additives, which have demonstrated remarkable enhancement in the properties of biodiesel [16]. Nano additives improve combustion characteristics, reduce exhaust and smoke emissions, and enhance engine performance [17]. For instance, by changing the concentrations of the nano additive, such as cerium oxide in the blended biodiesel, the specific fuel consumption (SFC) is decreased by 0.5 kg/kWh, mechanical efficiency is improved by 20%, and a decent change in the exhaust emissions has also been observed [18]. Similarly, the addition of 80 ppm of copper oxide NPs increased the brake thermal efficiency and reduced the specific fuel consumption at full load conditions. Moreover, these nano additives also help to decrease the emission of other harmful contents, including hydrocarbon (HC), carbon monoxide (CO), smoke, and NO_x_, at full load conditions [19]. In another study, when ZnO NPs were added to a dual fuel engine (biodiesel blend and hydrogen) at a concentration of 100 ppm, the emission of NO_x_ was reduced and the hydrogen flow rate was increased, leading to the enhancement of the performance of the engine [20]. These and several other studies have revealed that nano additives have a strong ability to minimize operational troubles and enhance ignition characteristics by lowering the oxidation temperature and improving the octane number of the fuel, which may lead to attaining fuel specification demands [21,22]. Therefore, apart from their use as heterogeneous catalysts during biodiesel production, metal oxide NPs, including ZnO NPs, can be applied as a catalyst to improve the performance of engines [23].

This study aims to prepare high-quality ZnO NPs using cow dung as fuel through a facile combustion technique, without using any external stabilizing agents or templates, in a short time. To demonstrate the catalytic properties, the as-prepared ZnO-GS NPs are applied to the transesterification of waste cooking oil (WCO) with methanol during the production of biodiesel. In addition, the catalytic property of ZnO-GS NPs is further tested in the enhancement of the fuel properties by adding different proportions of catalyst in biodiesel and diesel blends. The emission analysis of the engine is performed using an exhaust emission analyzer.

## 2. Results and Discussion

### 2.1. Characterization of the ZnO-GS Nanoparticles

**Figure 1:**
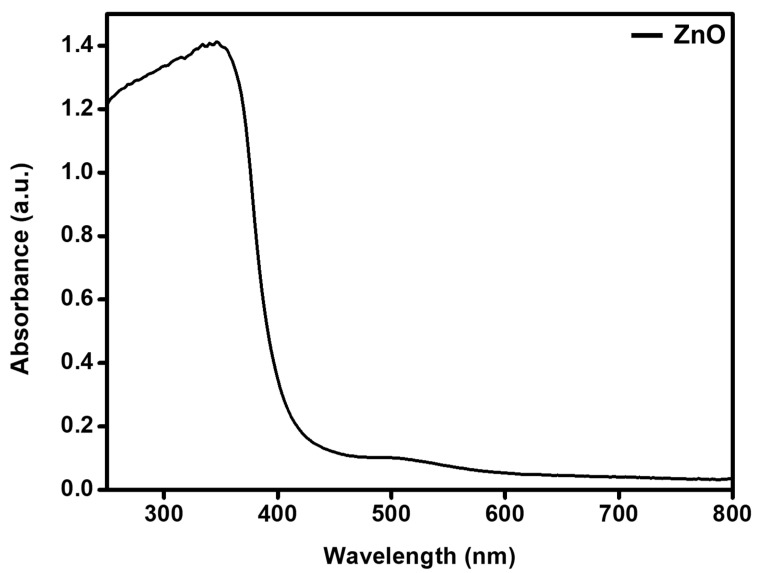
UV–Vis spectra of the prepared ZnO-GS nanoparticles.

UV-visible absorption of the as-synthesized ZnO-GS nanoparticles was measured at room temperature and the spectral data obtained are shown in Figure 1. The formation of ZnO-GS nanoparticles was confirmed by the formation of characteristic absorption maxima at 368 nm. This is due to the intrinsic bandgap absorption occurring by the transition of a valence band electron in 2p orbitals of oxygen to the 3d orbitals of Zn atoms (O_2p_→Zn_3d_), as reported earlier [24].

**Figure 2:**
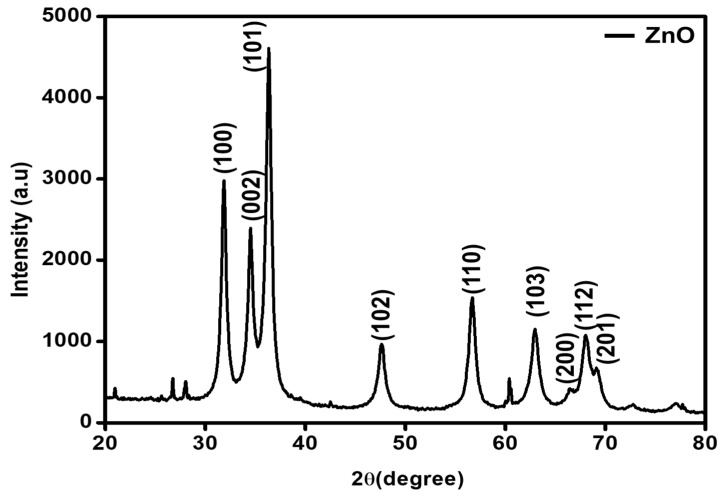
XRD diffractogram of as-synthesized ZnO-GS NPs.

Figure 2 reveals the diffractogram obtained for the ZnO-GS nanoparticles, which were obtained through the combustion method using cow dung as fuel. The diffracted peaks were generated at the following 2θ angles, viz.: 31.8°, 34.4°, 36.3°, 47.5°, 56.6°, 62.8°, 66.5°, 67.9°, and 69.1°can be indexed to (1 0 0), (0 0 2), (1 0 1), (1 0 2), (1 1 0), (1 0 3), (2 0 0), (1 1 2), and (2 0 1) crystal planes. All of these peaks are well-matched with the JCPDS card no. 36-1451 of the wurtzite crystal structure (space group P6_3_mc, a = 3.243 Å and c = 5.201 Å) [25]. The crystallite size along the (1 0 1) plane was found to be ~10 nm and the interplanar distance between the adjacent (1 0 1) planes was found to be ~0.25 nm, based on the calculation using the Debye–Scherrer formula and Bragg’s law, respectively.

**Figure 3:**
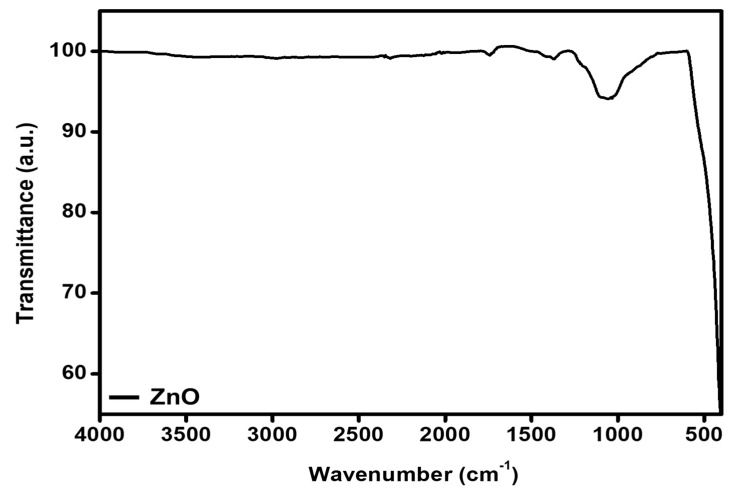
FT-IR spectrum of as-synthesized ZnO-GS NPs.

As-synthesized ZnO-GS nanoparticles were subjected to FT-IR spectral analysis between the frequency range of 400–4000 cm^−1^ and the obtained transmittance data are portrayed in Figure 3. The spectral data confirm the formation of ZnO nanoparticles with a sharp dip below the frequency of 500 cm^−1^, which is, according to earlier studies, related to ZnO nanoparticles [26]. However, the presence of other vibrational peaks—apart from the peak 415 cm^−1^—such as the broad peak at 1064 cm^−1^, indicates the presence of absorbed CO_2_, possibly from residues of cow dung, as the combustion fuel [27,28].

**Figure 4:**
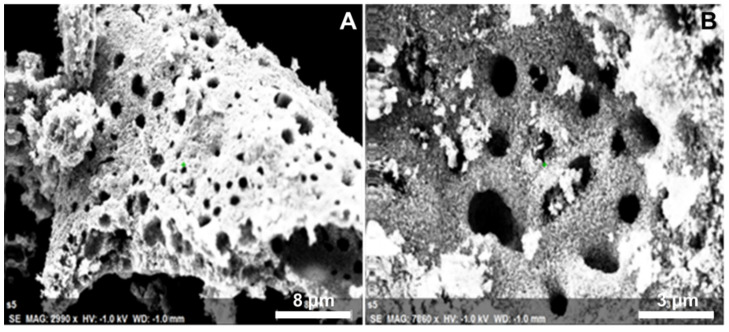
SEM images of the as-synthesized ZnO-GS NPs.

**Figure 5:**
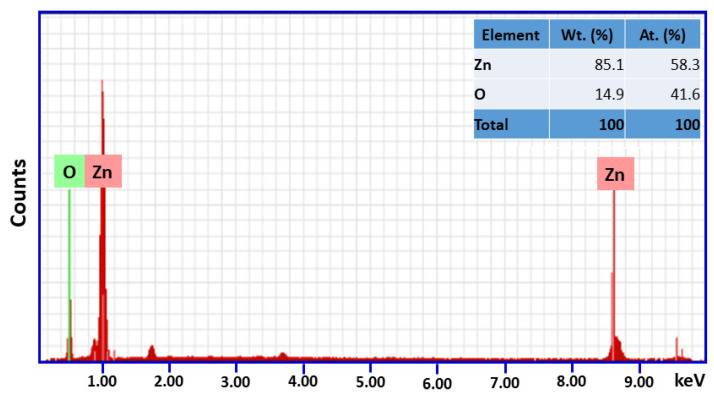
EDAX spectrum of ZnO-GS NPs.

The SEM images of varying magnifications are given in Figure 4. The images obtained of the as-synthesized ZnO-GS NPs reveal a porous surface with irregular morphology. The elemental composition of the prepared ZnO-GS nanoparticles is estimated using EDX spectroscopy and the percentage data obtained are given in Figure 5. It reveals that the synthesized heterostructure contains the desired elemental composition, i.e., Zn and O, and the percentage of elemental compositions are shown in the inset table.

N_2_ adsorption–desorption isotherm analysis was carried out to measure specific surface area and pore size distribution to discover the Brunauer–Emmett–Teller (BET) specific surface area and the Barrett–Joyner–Halenda (BJH) pore size distribution for the as-synthesized ZnO-GS nanoparticles. The shape of the isotherm in Figure S1 from Supplementary Materials indicates the aggregation of as-prepared ZnO-GS into sheets with several pores acting like slits. The type III isotherm formation indicates the weak interaction of the adsorbate on the surface of the as-prepared nanoparticle. The BET surface area of as-prepared ZnO-GS nanoparticles was found to be 10.3 m^2^/g. The pore size distribution curves in Figure S2 from Supplementary Materials confirmed the existence of small pores with diameters of less than 10 nm.

### 2.2. Physical Properties of D-COME

All tests of physical properties were carried out as per American Standards for Testing and Material (ASTM) in the laboratory at Christ University, Bangalore, India. The comparative results of the physical properties of commercial diesel, D-COME (as-prepared biodiesel) are listed in Table 1. The blend B20 with the further inclusions of ZnO-GS in blend B20, comprising 20 mg and 30 mg ZnO-GS, are termed B20-20 and B20-30, respectively. The results obtained were also compared with the chemically synthesized ZnO NPs, and the blends of 20 mg and 30 mg ZnO are termed B20-20-C and B20-30-C, respectively.

#### Brake Thermal Efficiency (BTHE) and Brake Specific Fuel Consumption (BSFC) Analysis

**Figure 6:**
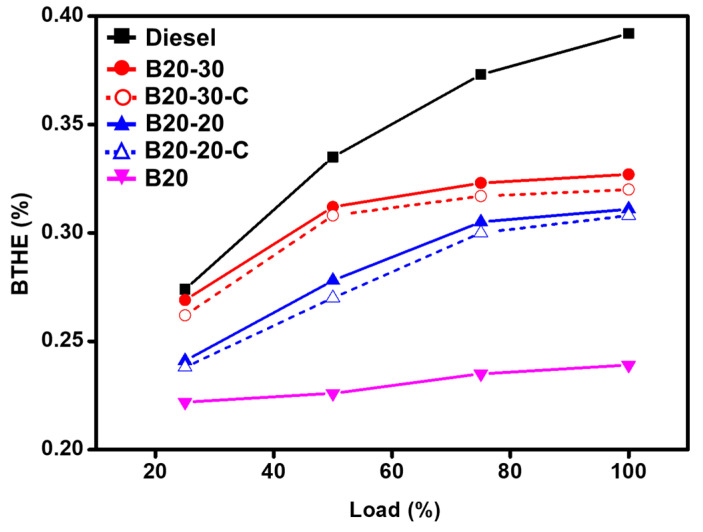
Comparative graphical illustration of brake thermal efficiency (BTHE) from diesel and blends vs. load (%) (with ZnO-GS and ZnO-C). B20 is D-COME, i.e., discarded cooking oil methyl ester, which is blended with 20% commercial diesel; B20-20 is the blend of B20 with 20 mg of ZnO-GS/C; B20-30 is the blend of B20 with 30 mg of ZnO-GS/C.

The impact of the blend B20 and the B20 blend with ZnO-GS nanoparticles, i.e., B20-20 and B20-30, on the engine performance, such as brake thermal efficiency (BTHE) and brake specific fuel consumption (BSFC), was analyzed. The values obtained from the analysis carried out are shown in Figure 6. It can be observed that the difference in brake thermal efficiency of commercial diesel is highest and the difference between it and the blend B20 is about 0.153%, indicating that the fuel efficiency of the system is much better for commercial fuel when compared to the blend B20. However, considerable improvements in the fuel efficiency of the blend B20 are observed when B20 is further blended by varying the amount of ZnO NPs; moreover, the blend with 30 mg, i.e., B20-30, displays the best fuel efficiency but is not comparable to commercial diesel. The dosage of ZnO from 20 mg to 30 mg shifts the performance of biodiesel towards diesel. Moreover, when the same studies were carried out with blends containing ZnO-C, it was found that the brake thermal efficiency (BTHE) was slightly lower than that of the green synthesized ZnO NPs, i.e., ZnO-GS.

The enhanced heat transfer characteristics, resulting in a faster combustion process, also higher oxygen content, help complete the combustion of fuel resulting in higher efficiency which can be attributed to the role played by the ZnO NPs due to an increase in surface area of the crystallite size of ZnO nanoparticles.

**Figure 7:**
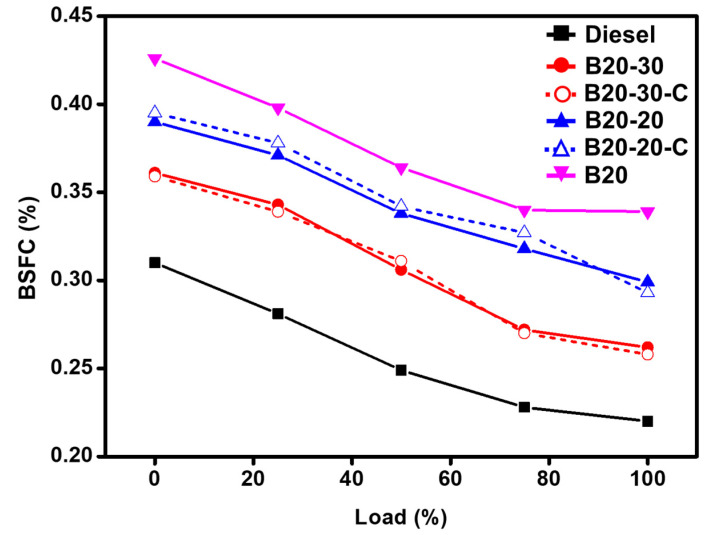
Comparative graphical illustration of brake specific fuel consumption (BSFC) diesel and blends vs. load (%) (with ZnO-GS and ZnO-C). B20 is D-COME, i.e., discarded cooking oil methyl ester, which is blended with 20% commercial diesel; B20-20 is the blend of B20 with 20 mg of ZnO-GS/C; B20-30 is the blend of B20 with 30 mg of ZnO-GS/C.

Furthermore, the brake-specific fuel consumption (BSFC) values for the blends, indicating the engine performance characteristics required for the conversion of fuel consumption to work, were also obtained and compared with the commercial diesel (Figure 7). It can be seen that the fuel consumption is higher with blend B20 when compared to commercial diesel; however, by adding varying amounts of ZnO NPs to the blend B20, i.e., B20-20 and B20-30, the brake specific fuel consumption (BSFC) values can be reduced significantly. This could be because of higher oxygen content or reduced ignition delay that enhances the combustion properties. The enhanced combustion efficiency leads to an increase in the momentum of fuel and propagation of fuel, resulting in cloud-like emulsified moisture during the injection. Moreover, when the same studies were carried out with blends containing ZnO-C, it was found that the brake specific fuel consumption (BSFC) values were almost the same as that of the green synthesized ZnO NPs, i.e., ZnO-GS.

### 2.3. Carbon Monoxide (CO) Emission

**Figure 8:**
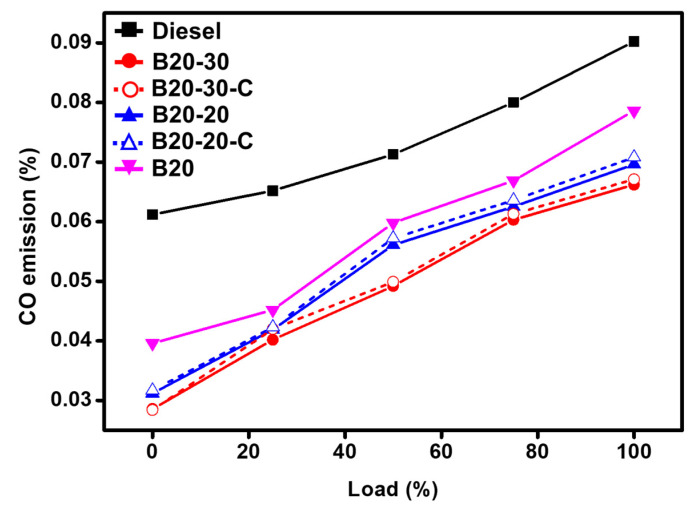
Comparative graphical illustration of emission of CO from diesel and blends vs. load (%) (with ZnO-GS and ZnO-C). B20 is D-COME, i.e., discarded cooking oil methyl ester, which is blended with 20% commercial diesel; B20-20 is the blend of B20 with 20 mg of ZnO-GS/C; B20-30 is the blend of B20 with 30 mg of ZnO-GS/C.

The combustion of fuel in a shorter time and lack of oxygen leads to the formation of carbon monoxide. It was found that commercial diesel yielded 0.09% of CO, while blended B20 yielded 0.08%. However, in the blends with ZnO nanoparticles, i.e., B20-20 and B20-30, the ZnO NPs behaved as oxygen donating catalysts and improved the oxidation of CO, which is evident with the 0.07% and 0.06% of CO obtained, respectively. The metallic ZnO NPs were found to be favorable with respect to its effects on emission reduction and engine performance enhancement. Moreover, with blends containing ZnO-C, i.e., B20-20-C and B20-30-C, it is found that the CO emission values were almost the same as those of the green synthesized ZnO NPs, i.e., ZnO-GS. The graphical illustration of results obtained is given in Figure 8.

### 2.4. Carbon Dioxide (CO_2_) Emission

As observed from the previous study, the blends with higher ZnO NPs, i.e., B20-20 and B20-30, yielded lower CO emissions.

However, the studies of the impact of ZnO-GS and ZnO-C NPs blended concentration in B20 on the emission of CO_2_ reveal that the blends with higher ZnO NPs, i.e., ZnO-GS and ZnO-C NPs, yield an increase in CO_2_ emission, this could be due to the complete combustion of hydrocarbons and CO, which produces carbon dioxide and water as combustion products. An amount of 9.1% of CO_2_ was obtained from the use of commercial diesel, while 13.7% of CO_2_ was obtained from the use of B20-30. The increased concentration of carbon dioxide in emissions also indicates the lesser emission of carcinogenic carbon monoxide, by converting it into carbon dioxide due to the presence ZnO-GS NPs in the blend B20, which acts as an oxidizing agent. Moreover, with blends containing ZnO-C, it was found that the CO_2_ emission values were almost the same as those of the green synthesized ZnO NPs, i.e., ZnO-GS.

**Figure 9:**
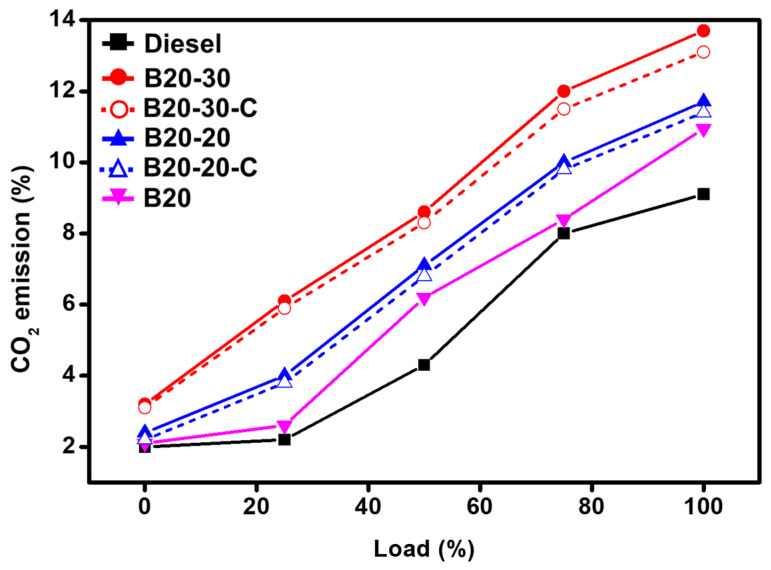
Comparative graphical illustration of emission of CO_2_ from diesel and blends vs. load (%) (with ZnO-GS and ZnO-C). B20 is D-COME, i.e., discarded cooking oil methyl ester, which is blended with 20% commercial diesel; B20-20 is the blend of B20 with 20 mg of ZnO-GS/C; B20-30 is the blend of B20 with 30 mg of ZnO-GS/C.

Moreover, it can be said that the addition of nanoparticles results in a reduced ignition delay period, enhanced the calorific value, and oxidation rates leading to a complete and cleaner combustion. The pictorial representation emission pattern obtained are plotted in Figure 9.

### 2.5. Nitrogen Oxide (NOx) Emission

**Figure 10:**
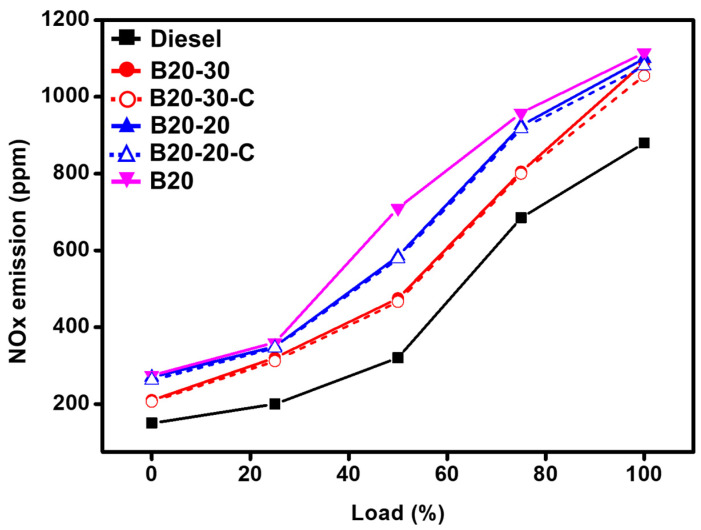
Comparative graphical illustration of emission of NO_x_ vs. load (%) (with ZnO-GS and ZnO-C). B20 is D-COME, i.e., discarded cooking oil methyl ester, which is blended with 20% commercial diesel; B20-20 is the blend of B20 with 20 mg of ZnO-GS/C; B20-30 is the blend of B20 with 30 mg of ZnO-GS/C.

The NO_x_ emission from fuels is due to the presence of nitrogen compounds along with due to higher oxygen content and increased combustion chamber temperature. The blended B20 emits NO_x_ gases of 1115 ppm, which is much more than the commercial diesel, which emits 880 ppm. However, the further addition of ZnO NPs, i.e., B20-20 and B20-30, have been found to mildly minimize NO_x_ emissions to 1115 ppm and 1100 ppm, respectively; this could be attributed to the decrease in combustion duration, affirmative effect, and advanced catalytic effect, which breaks NO_x_ into simple nitrogen (Figure 10).

### 2.6. Hydrocarbon (HC) Emission

**Figure 11:**
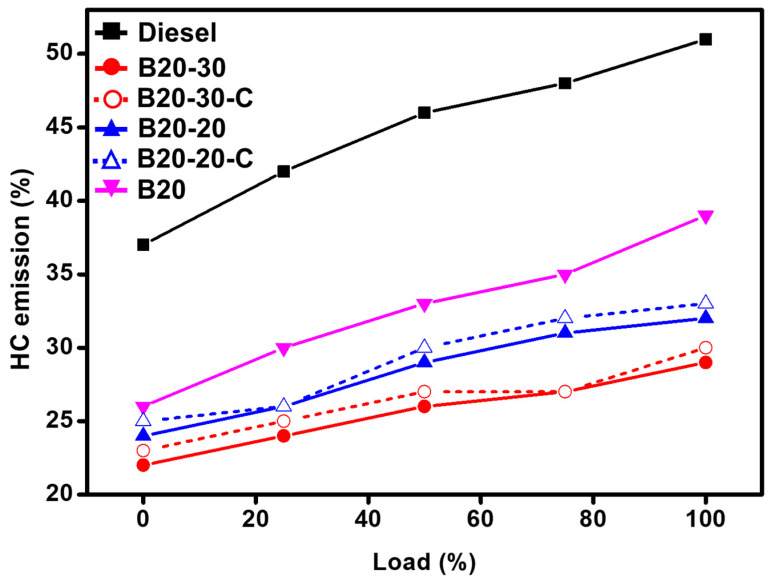
Comparative graphical illustration of emission of hydrocarbons (HC) vs. load (%) (with ZnO-GS and ZnO-C). B20 is D-COME, i.e., discarded cooking oil methyl ester, which is blended with 20% commercial diesel; B20-20 is the blend of B20 with 20 mg of ZnO-GS/C; B20-30 is the blend of B20 with 30 mg of ZnO-GS/C.

The reason behind the hydrocarbon (HC) emission from fuels is due to incomplete combustion of fuel fragments. Commercial diesel is found to yield 51% HC; however, the B20 blend yields 39% HC. The addition of ZnO NPs to B20 as the blends B20-20 and B20-30, reveals that the HC emission can be drastically reduced to up to 32% and 29%, respectively. The reduction in HC emission could be due to the catalytic activity of ZnO, which enables the complete oxidation of the HC in the fuel fragments. A relative graphical illustration is given in Figure 11.

It is important to note that there is a possibility of the presence of a certain amount of ZnO NPs in exhaust gases, which can be adsorbed into the catalytic convertor of the automobile in a real-life situation; however, this aspect warrants further study.

## 3. Materials and Methods

### 3.1. Materials

With no further purification, analytical grade Zinc nitrate hexahydrate Zn(NO_3_)_2_·6H_2_O (Sigma-Aldrich, MO, USA) is used. Cow dung is collected and dried under sunlight for 15 days is used as fuel. Discarded cooking oil is collected from several residences and pretreated before being used for transesterification.

### 3.2. Green Synthesis of ZnO Nanocatalyst (ZnO-GS)

A calculated amount of (Zn(NO_3_)_2_·6H_2_O was dissolved in 8 cm^3^ of distilled water and 0.9 g of fresh cow dung was added under constant stirring for 20 min. Subsequently, the mixture was kept in a muffle furnace at 400 °C. After 10 min a pale-yellow powder was obtained, which was annealed at the same temperature for 3 h. ZnO-C NPs were also synthesized by the precipitation method [30], wherein a base was added drop-wise to Zinc salt solution until the pH of the solution reaches 9 and can be used for transesterification and emission analysis.

### 3.3. Synthesis of Discarded Cooking Oil Methyl Ester (D-COME)

Discarded cooking oil was collected from several residences and mixed well and pre-treated. The optimization of transesterification was carried out by changing the concentration of methanol, the amount of the ZnO-GS catalyst, and the temperature (Table 2).

The molar ratio of alcohol to oil is one of the most significant factors affecting conversion efficiency, the yield of biodiesel, as well biodiesel production cost. Additionally, since the stoichiometric molar ratio of alcohol to oil for the transesterification is 3:1 and the reaction is reversible, higher molar ratios are required to increase the miscibility and enhance the contact between the alcohol molecule and the triglyceride. In practice, to shift the reaction towards completion the molar ratio should be higher than that of the stoichiometric ratio. To further break the glycerin–fatty acid linkages during the transesterification of triglycerides to biodiesel, excess methanol is required. Therefore, higher alcohol to oil molar ratios gave rise to greater alkyl ester conversion in a shorter time. Moreover, an increase in the amount of alcohol to oil increases biodiesel yield and biodiesel purity. On the contrary, inedible oils like Pongamia and neem require more alcohol to give maximum ester yield, possibly due to the higher viscosity of inedible oil than edible oils. However, when compared to edible oil, ester content yield was small in inedible oil, but glycerol yield was larger in inedible oil when compared to edible oil. The key variables affecting transesterification are reaction time, alcohol to oil molar ratios, reaction temperatures, catalysts, water contents, and free fatty acids levels in fats and oils.

In this current study, the temperature was varied from 55 °C to 70 °C. It was observed that at 65 °C the reaction yielded a 97.2% transesterification product, which is the highest among the series of reactions carried out at various reaction temperatures, with constant stirring time for 90 min. Therefore, the temperature was maintained at 65 °C for further studies. Methanol variation was carried out by employing different volumes of methanol, ranging between 100 mL to 175 mL with a constant amount of the catalyst ZnO-GS at 65 °C. Studies showed that reactions in which 150 mL of methanol was utilized yielded 97.2% of biodiesel, i.e., D-COME. Hence, 150 mL of methanol was maintained for detailed studies.

**Figure 12:**
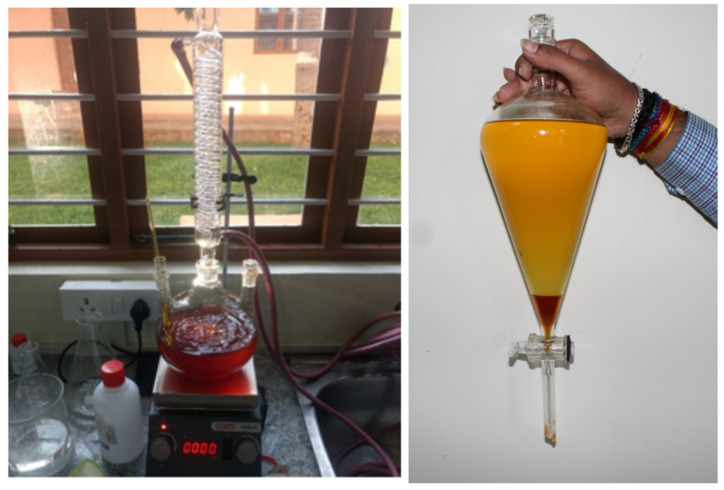
Experimental set up for the transesterification of discarded cooking oil and isolation of D-COME.

As reported in various studies, the amount of catalysts plays a vital role in any transformation reaction, and the optimization of the amount of catalysts was studied by varying the amount of ZnO-GS NPs from 125 mg to 200 mg. It was observed that the transesterification product increases with the increase of the amount of catalyst up to 175 mg; however, a further increase to 200 mg did not yield a significant improvement in the amount of product obtained. Hence, it can be said that 175 mg of the catalyst is the optimum amount of catalyst, while 150 mL of methanol at 65 °C is the optimum condition for the transesterification of 1 L of discarded cooking oil to obtain the desired biodiesel (Figure 12). A comparative reaction was carried out using the ZnO NPs, i.e., ZnO-C, prepared by the chemical method under the optimized conditions and was observed. Of the conversion product, ~97% was obtained, indicating that there was no loss of catalytic efficiency in the ZnO NPs (i.e., ZnO-GS) due to the green synthetic protocol employed for their preparation.

### 3.4. Blending of D-COME

To further the above catalytic studies, ZnO-GS was employed as a blend. D-COME was mixed with commercial diesel at 20% by volume. This blend is termed B20, as standardized by earlier studies [31]. Furthermore, this blend was further combined with varying amounts of ZnO-GS NPs, i.e., 20 mg and 30 mg, which were termed B20-20 and B20-30, respectively, using an ultrasonication bath, and subsequently used as fuels for engine experiments. Similar blends were created by employing the chemically synthesized ZnO NPs, i.e., ZnO-C, and the blends were labeled B20-20-C and B20-30-C, which contain varying amounts of ZnO-C NPs, i.e., 20 mg and 30 mg, respectively.

### 3.5. Characterization of ZnO-GS Nanocatalyst

The prepared nanostructures were characterized by XRD, FT-IR, FESEM, and TEM. The XRD characterization were carried out using a Bruker diffractometer, using Cu Kα (λ = 1.5406 Å) as the X-ray source. The spectral characterization was carried out using a Perkin–Elmer UV–vis spectrometer for UV-vis and a Bruker IFS 66 v/S spectrometer for FT-IR. Microscopic analysis, such as SEM, was carried out to understand the surface morphology and particle size using a field emission scanning electron microscope (FESEM), and the TEM images were recorded with a transmission electron microscope, JEOL JEM2100 PLUS, operating at 200 kV accelerating voltage.

### 3.6. Experimental Setup and Procedure

**Figure 13:**
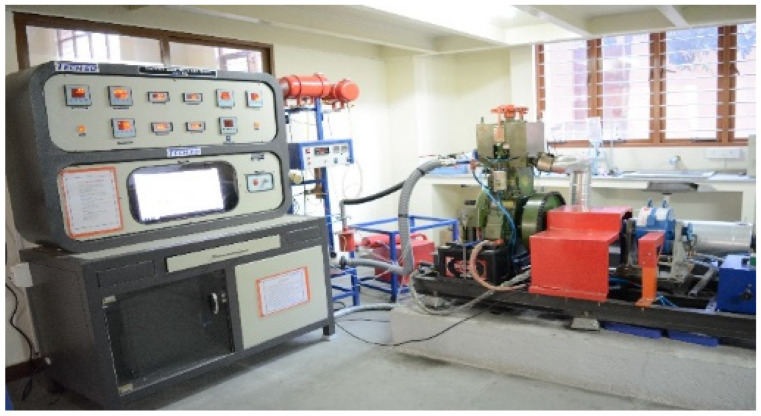
Digital image of diesel engine (Make: Kirloskar).

**Figure 14:**
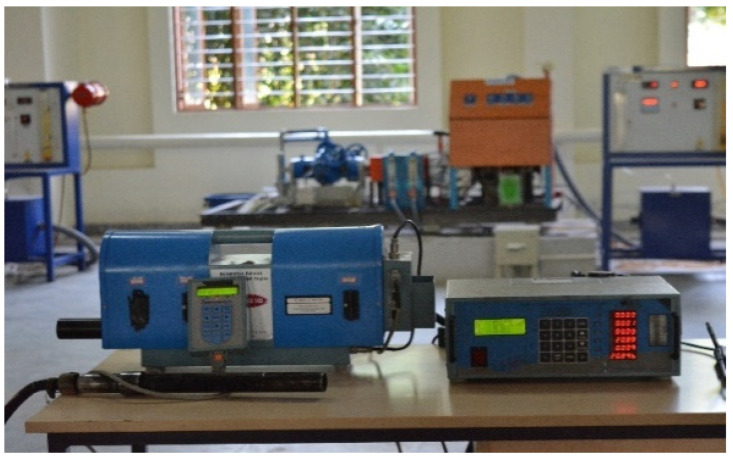
Digital image Indus scientific exhaust gas analyzer.

The performance tests were carried out on a Kirloskar TV1 four stroke single cylinder, water-cooled compression ignition engine (Figure 13). An eddy current dynamometer was used for engine loading. Sensors were placed at required junctions to calculate the temperatures. A rotameter was used for measuring the engine jacket water and cooling water flow rates. The detailed specification of the engine is given in Table 3. An Indus exhaust gas analyzer (Figure 14) was used for measuring emission parameters.

## 4. Conclusions

In conclusion, we reported the green synthesis of ZnO-GS NPs, using cow dung as fuel, where ZnO-GS NPs were employed as transesterification catalysts for the preparation of biodiesel through the reaction of used cooking oil to obtain biodiesel (D-COME), which was then blended with 20% commercial diesel, resulting in B20. Emission analysis of the heterogeneous blend of B20, prepared by blending 30 mg of as-prepared ZnO-GS NPs with B20 i.e., B20-30, reduced the emission of greenhouse gases considerably, as well as improving fuel efficiency. Lastly, it can be stated that B20-30 could be safely used without any modifications to the engine and without any operational difficulty in order to obtain an increase in brake thermal efficiency.

## Figures and Tables

**Table 1:** Physical properties of commercial diesel and D-COME.

S. No.	Property	Diesel [29]	D-COME
1	Calorific Value (kJ/kg)	42,000	39,216
2	Flash Point [°C]	52–96	182
3	Fire Point [°C]	62–106	134
4	Density at 15 °C [g/m^3^]	824	925
5	Viscosity [mm^2^/s]	1.2–2	4.56
6	Octane Number	48	55

**Table 2:** Optimization of conditions for the preparation of D-COME.

Variation		Yield (%)
Temperature (°C) Conditions: Catalyst ZnO-GS 175 mg; Methanol 150 mL; Time 90 min	55	69.5
	60	82.3
	65	97.2 (96.8)
	70	96.6
Methanol (mL) Conditions: Catalyst ZnO-GS 175 mg; Temperature 65 °C; Time 90 min	100	82.5
	125	92.5
	150	97.2
	175	97.3
ZnO-GS (mg) Conditions: Methanol 150 mL; Temperature 65 °C; Time 90 min	125	81.4
	150	90.3
	175	97.2
	200	97.5

**Table 3:** Engine specifications (Make: Kirloskar).

Parameters	Value
Bore (mm)	80
Stroke Length (mm)	110
Connecting Rod Length (mm)	234
Displacement Volume (cc)	552
Rated Speed (RPM)	1500
Max Power (HP)	5
Max Torque (Nm)	24
Compression Ratio	16.5

## Data Availability

Data contained within the article.

## Supplementary Materials

The following supporting information can be downloaded at: https://www.mdpi.com/article/10.3390/molecules27092845/s1, Figure S1: N_2_ adsorption-desorption isotherm of ZnO-GS nanoparticles; Figure S2: Pore size distribution of ZnO-GS nanoparticles.

## Sample Availability

Samples of the compounds are/are not available from the authors.
