# Supplementary material for: Green Synthesized ZnO Nanoparticles as Biodiesel Blends and Their Effect on the Performance and Emission of Greenhouse Gases
Source: Molecules. 2022 Apr 29;27(9):2845. doi: 10.3390/molecules27092845 (PMC9105191; doi:10.3390/molecules27092845)
Supplement: Supplementary file 1 [file molecules-27-02845-s001.zip › molecules-1638129-supplementary.pdf]

molecules

MDPI

Supplementary

Green Synthesized ZnO Nanoparticles as Biodiesel Blends and its Effect on Performance and Emission of Greenhouse Gases

Kiran Kavalli [1], Gurumoorthy S Hebbar [1], Jayachamarajapura Pranesh Shubha [2,*], Syed Farooq Adil [3,*], Mujeeb Khan [3], Mohammad Rafe Hatshan [3], Adibah Mukhlid Almutairi [3] and Baji Shaik [4]

1 Department of Mechanical & Automobile Engineering, Christ University, Bangalore, India; kiran.k@christuniversity.in (K.K.); gshebbar@gmail.com (G.S.H.)
2 Department of Chemistry, Don Bosco Institute of Technology, Mysore Road, Bangalore-560074, India
3 Department of Chemistry, College of Science, King Saud University, P.O.Box 2455, Riyadh-11451, Saudi Arabia; kmujeeb@ksu.edu.sa (M.K.); mhatshan@ksu.edu.sa (M.R.H.); adeba@ksu.edu.sa (A.M.A.)
4 Department of Advanced Materials Engineering for Information & Electronics, Kyung Hee University, 1732 Deogyeong-daero, Giheung-gu, Yongin-si 446-701, Gyeonggi-do, Korea; shaikbaji2@khu.ac.kr (B.S)
* Correspondence: shubhapranesh@gmail.com; sfadil@ksu.edu.sa

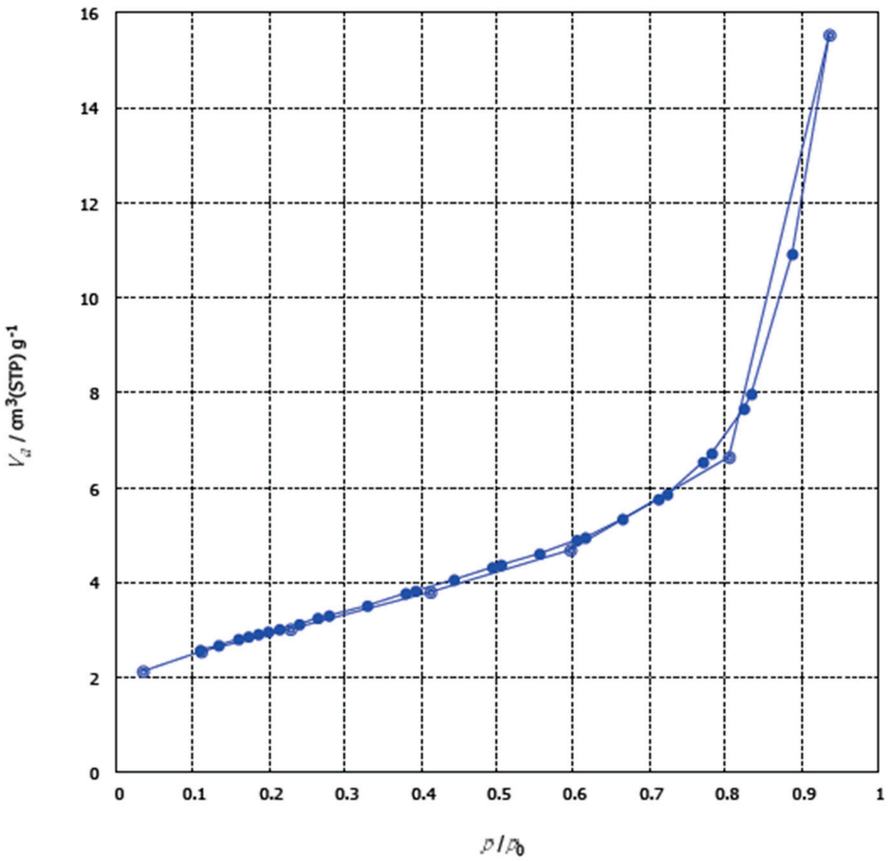

Figure S1. N_2 adsorption-desorption isotherm of ZnO-GS nanoparticles

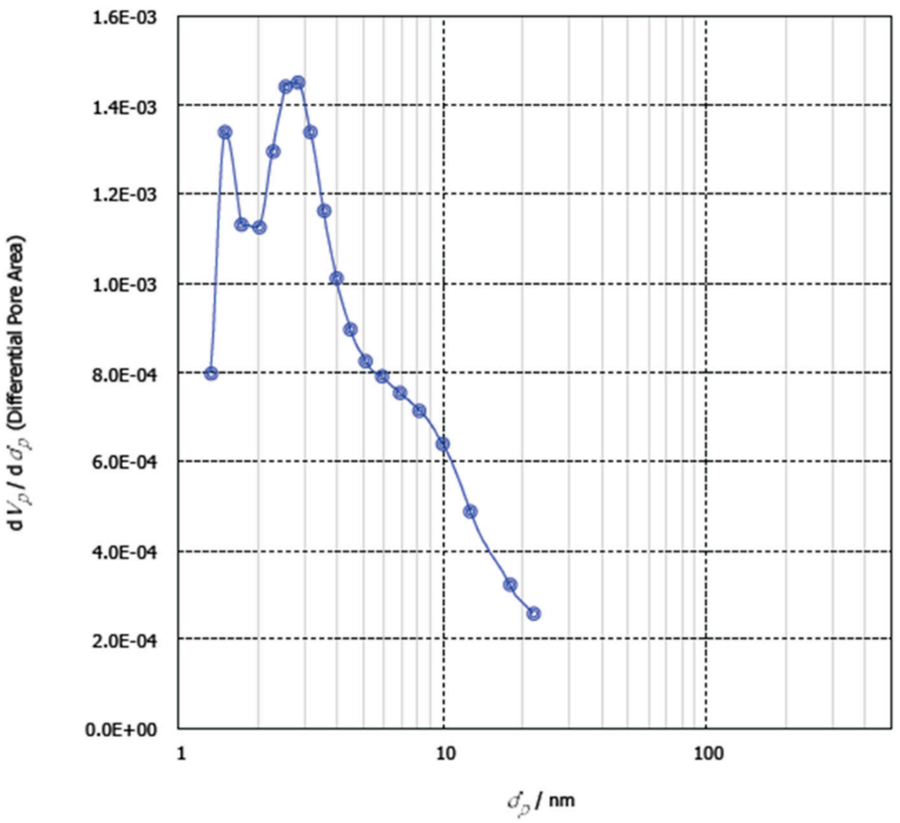

Figure S2. Pore size distribution of ZnO-GS nanoparticles
